# Floating Ricobendazole Delivery Systems: A 3D Printing Method by Co-Extrusion of Sodium Alginate and Calcium Chloride

**DOI:** 10.3390/ijms23031280

**Published:** 2022-01-24

**Authors:** Giovanni Falcone, Juan P. Real, Santiago D. Palma, Rita P. Aquino, Pasquale Del Gaudio, Emilia Garofalo, Paola Russo

**Affiliations:** 1 Department of Pharmacy, University of Salerno, 84084 Fisciano, Italy; gifalcone@unisa.it (G.F.); aquinorp@unisa.it (R.P.A.); pdelgaudio@unisa.it (P.D.G.); 2 PhD Program in Drug Discovery and Development, University of Salerno (SA), 84081 Fisciano, Italy; 3 Unidad de Investigación y Desarrollo en Tecnología Farmacéutica (UNITEFA), CONICET, Córdoba X5000HUA, Argentina; juan.real@unc.edu.ar (J.P.R.); sdpalma@unc.edu.ar (S.D.P.); 4 Departamento de Ciencias Farmacéuticas, Facultad de Ciencias Químicas, Universidad Nacional de Córdoba, Ciudad Universitaria, Córdoba X5000HUA, Argentina; 5 Department of Industrial Engineering, University of Salerno, 84081 Fisciano, Italy; egarofal@unisa.it

**Keywords:** semi-solid extrusion 3D printing, alginate ionotropic gelation, floating DDS

## Abstract

At present, the use of benzimidazole drugs in veterinary medicine is strongly limited by both pharmacokinetics and formulative issues. In this research, the possibility of applying an innovative semi-solid extrusion 3D printing process in a co-axial configuration was speculated, with the aim of producing a new gastro-retentive dosage form loaded with ricobendazole. To obtain the drug delivery system (DDS), the ionotropic gelation of alginate in combination with a divalent cation during the extrusion was exploited. Two feeds were optimized in accordance with the printing requirements and the drug chemical properties: the crosslinking ink, i.e., a water ethanol mixture containing CaCl_2_ at two different ratios 0.05 M and 0.1 M, hydroxyethyl cellulose 2% *w*/*v*, Tween 85 0.1% *v*/*v* and Ricobendazole 5% *w*/*v*; and alginate ink, i.e., a sodium alginate solution at 6% *w*/*v*. The characterization of the dried DDS obtained from the extrusion of gels containing different amounts of calcium chloride showed a limited effect on the ink extrudability of the crosslinking agent, which on the contrary strongly influenced the final properties of the DDS, with a difference in the polymeric matrix toughness and resulting effects on floating time and drug release.

## 1. Introduction

Benzimidazole composites, due to their wide spectrum of action, good tolerability, and low toxicity are at present the first-choice drugs for the treatment of helminthic parasites such us nematode and trematode in domestic and livestock animals [1,2]. The pharmacokinetic properties of these anthelmintic drugs, in particular the rate and extent of gastrointestinal absorption, are strongly influenced by some extrinsic parameters, i.e., species, dosage, and function of the esophageal groove reflex [3]. For this reason, the control and the improvement of the benzimidazole pharmacokinetic profile represent an important challenge to allow for efficacious parasite control in animals [4,5].

To achieve this goal, for more than three decades the benzimidazole backbone underwent to chemical modifications to develop several subclasses: triazoles, probenzimidazoles and methylcarbamates [6]. However, these compounds were characterized by a very low water solubility that limits the drug’s bioavailability, creating a technological challenge [7]. For example, the oral administration of ricobendazole, the albendazole sulfoxide with the highest water solubility among the derivatives, is almost limited to suspension, paste, or bolus [8]. To maximize the drug contact time with the infected tissues and therefore the antiparasitic efficacy, modified drug delivery systems have been studied [9]. Among these products, gastro-retentive drug delivery systems (GRDDS) can potentially be the most suitable forms in accordance with the technological and pharmacokinetic requirements of methylcarbamate anthelmintic drugs [10]. GRDDS are oral dosage forms able to resist gastric emptying, remaining in the stomach for a long period of time thanks to different physical processes, such as bioadhesion, swelling, or floating [9,11].

For GRDDS development, some innovative production processes have been evaluated, such us laminar jet break-up, spray drying, and more recently 3D printing [12,13]. In fact, 3D printing technologies, a set of additive manufacturing techniques characterized by a layer-by-layer production process [14], have been widely used in several fields connected with human healthcare, i.e., tissue engineering, medical implant development, and preclinical drug development stages [15,16]. Choi et al. described the combination of gas foaming and FDM 3D printing to produce a scaffold that could fulfil both mechanical and cell availability requirements [17]. Thanks to the manufacturing flexibility and high resolution, the interest in the application of 3D printing technologies in pharmaceutical production has grown exponentially over the last five years, as confirmed by the large number of published articles [18,19,20,21]. For example, thanks to 3D printing technologies, innovative pulsatile drug delivery systems, as described by Dumpa et al. [22], or biodegradable patches with a particular infill able to control drug release [23] have been developed. Focusing on the production of floating systems, two different 3D printing technologies were mainly tested, i.e., fused deposition modelling and semi-solid extrusion, with the aim of enhancing the gastric residence time varying across several parameters, such as the number of layers and the infill percentage, or the ink–gel composition [24,25,26,27].

In previous research, the development of a new floating 3D-printed DDS based on alginate, customizable in shape and/or in drug amount, was described. In particular, a co-axial extrusion system was set up to exploit the ionotropic gelation of alginate during the printing process to extrude hollow filaments [28]. Following the potential advantages of ricobendazole gastric release, in the present study the innovative calcium-alginate floating system previously described was applied to this veterinary drug to obtain new GRDDS.

## 2. Results

### 2.1. Alginate Ink and Crosslinking Ink Preparation

By direct incorporation of ricobendazole (RBZ) in the aqueous crosslinking gel, a biphasic system was obtained due to the drug’s poor solubility in water, which caused a nozzle obstruction during the printing. Following the information proposed by Wu et al. about the solubility and lipophilicity of RBZ, some different hydroalcoholic mixtures were tested [8]. Particular attention was paid to the water/ethanol mixture, which was evaluated in three different ratios from 90/10 to 70/30 (data not shown). In particular, crosslinking ink with homogeneous drug incorporation and good extrudability was obtained using H2O/EtOH 80/20 as solvent (HEC 2% *w*/*v*, RBZ 5% *w*/*v*, and Tw 0.1% *v*/*v*) and assaying two different concentrations of calcium chloride (0.1 and 0.05 M).

### 2.2. Rheological Studies

To understand the physical characteristics of gels and consequently relate them to the semi-solid extrusion printing requirements, the rheological behavior of both feeds was analyzed [29,30]. In particular, attention was focused mainly on the crosslinking feed to highlight the impact of ethanol, calcium, and ricobendazole on the gel properties.

**Figure 1:**
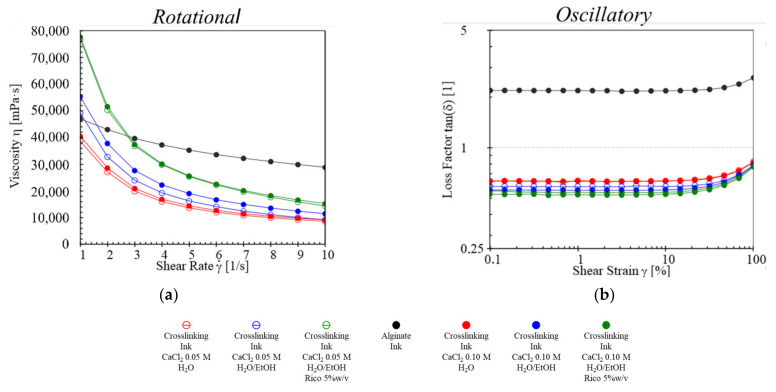
Results from rotational test (**a**) and amplitude sweep test (**b**) for crosslinking and alginate inks.

Through a rotational test (Figure 1a), it was possible to observe a constant slight decrease in viscosity at all shear rate values for the alginate ink curve (black line); this was different from all crosslinking inks, which showed a rapid decrease in viscosity. Moreover, the increase in calcium concentration led to a minimum increase in the viscosity of the crosslinking feed both in water and hydroalcoholic gels (filled markers with 0.10 M of calcium vs. empty markers with 0.05 M of calcium). The alcohol content led to an increase in gel viscosity (blue markers vs. red ones), which further showed a 60% growth after the addition of the drug (green markers). The results obtained from the oscillatory test (Figure 1b) helped to highlight the key variations between alginate and crosslinking inks, showing a loss factor higher than one and around 0.5, respectively [31]. These differences were taken into consideration during the set-up of the print flow rates for alginate and crosslinking inks. According to the properties of both alginate and crosslinking inks [32], the flow rates were fixed at 50 µL/min and at 100 µL/min, respectively.

### 2.3. Design, Development, and Production of DDS

**Figure 2:**
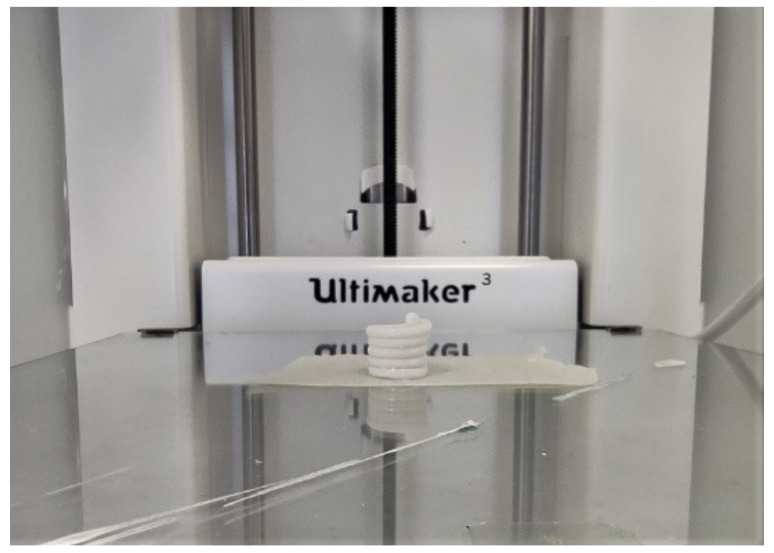
Photo of DDS immediately after printing.

The DDS produced (Figure 2) showed very low variations in mass, both after printing and after drying, underlining the high reproducibility of the co-axial printing process, independently from the crosslinking agent concentration used (Table 1), the latter being very important in shape retention after the drying process. DDS0.05_R5, in fact, did not maintain its shape during the drying, as evidenced by a final height of only 33% compared to the height of the model after printing (Table 1).

### 2.4. FT-IR and DSC Analysis

**Figure 3:**
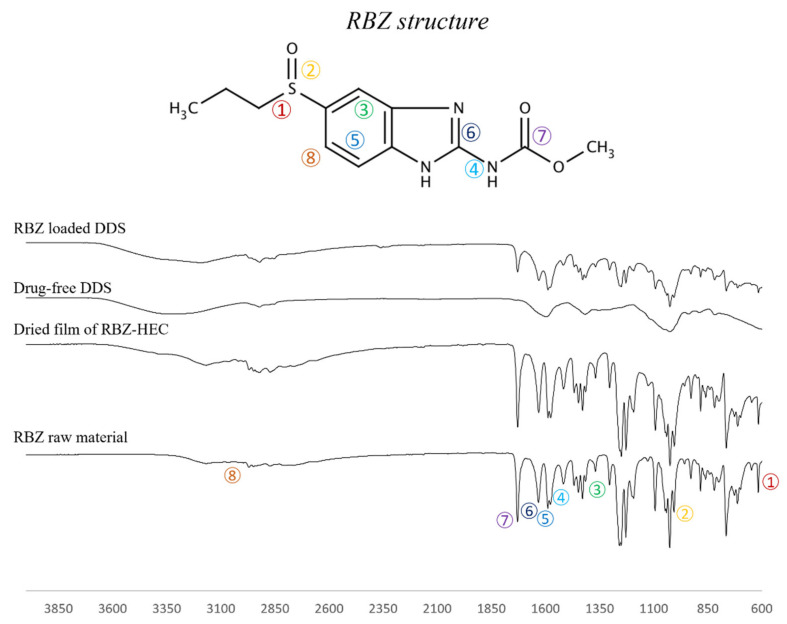
FT-IR spectrum of RBZ raw material, dried film obtained from RBZ-HEC gel, drug-free DDS and RBZ loaded DDS; RBZ structure, with schematic representation of the chemical bonds, was reported to allow for data analysis.

In accordance with the literature [33], all peaks related to the benzimidazolic rings i.e., C-C at 1304 cm^−1^ (peak 3), C=C stretch at 1586 cm^−1^ (peak 5), C-N at 1516 cm^−1^ (peak 4), C=N at 1633 cm^−1^ (peak 6), C-H from 3050 to 3171 cm^−1^(large bandwidth 8), as well as the stretch of the C=O of amidic group around 1729 cm^−1^ (peak 7), the sulfuric group C-S at 648 cm^−1^ (peak 1), and S=O at 1006 cm^−1^ (peak 2), which distinguish the RBZ from the other benzimidazolic compounds, were identified without modification in the spectrum of drug-loaded DDS (Figure 3).

**Figure 4:**
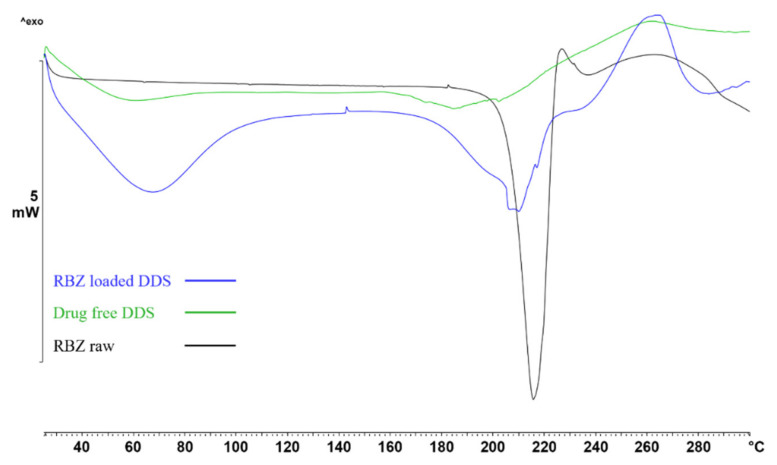
Thermographs of RBZ raw material (black line), drug-free DDS (green line), and RBZ-loaded DDS (blue line).

Through DSC analysis (Figure 4), the RBZ raw material exhibited an endothermic band from 207 to 224 °C due to the melting of the API polymorphs [34] (black line in Figure 4). DDS (green and blue lines in Figure 4) exhibited a broad peak at temperatures lower than 100 °C due to the loss of water, and in the region around 200 °C. The peaks corresponding to the breakage of calcium–carboxylate bonds in the formed egg-box structure of the alginate polymers are clearly visible [35] in the drug-free DDS (green line) and in the drug-loaded systems (blue line), but with different temperatures and peak broadness. The variation in temperature and profile of the drug melting peak and of the breakage of calcium–carboxylate bonds in the polymer might be correlated with a drug–polymer interaction.

### 2.5. Morphological Analysis

**Figure 5:**
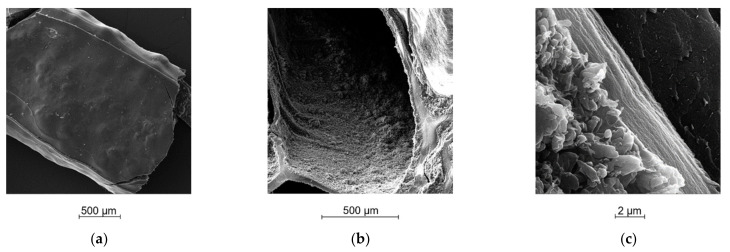
SEM acquisition of: (**a**) external view of single layer after drying; (**b**) a cross-section view of DDS; (**c**) a longitudinal-section view of DDS.

**Figure 6:**
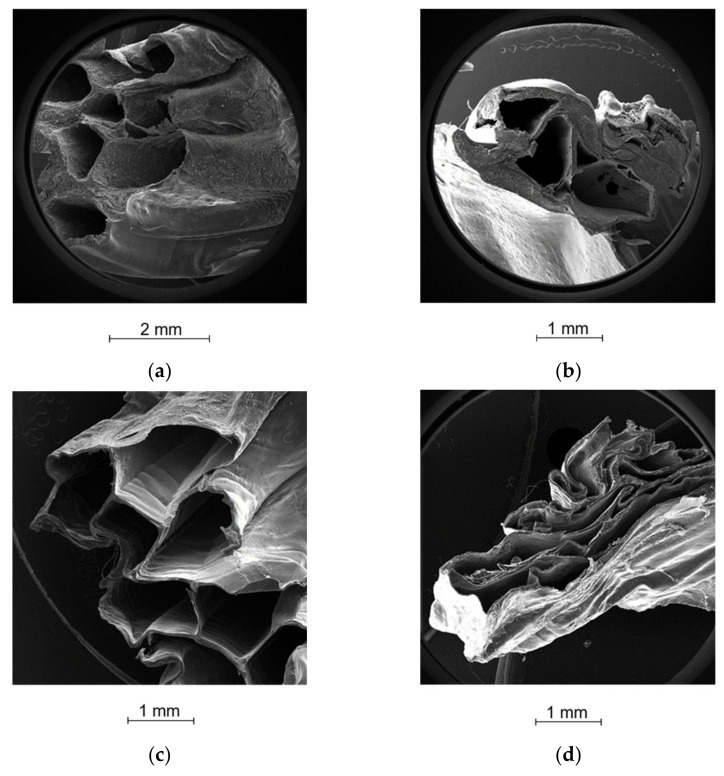
Cross-section SEM acquisition of (**a**) dried DDS0.10_5; (**b**) dried DDS0.05_5; (**c**) DDS0.10_5 after dissolution and re-drying; (**d**) and DDS0.05_5 after dissolution and re-drying.

SEM acquisitions Figure 5a,b allowed for highlighting the hollow filament formation and the homogeneous distribution of RBZ inside the alginate shell. In Figure 5c it is possible to observe the filament stratification from inside out: RBZ in crystal form, HEC foil, and the shell of calcium alginate on the bottom. Moreover, looking at a cross section of the printout (Figure 6), DDS0.05_R5 produced from gel with the lowest calcium amount showed an internal collapse after drying (Figure 6b) that led to the formation of RBZ niches and a dramatic reduction in empty spaces (Table 1). On the other hand, the DDS0.1_R5 after drying showed good shape retention with a defined hollow filament and a more uniform distribution of API next to the alginate shell (Figure 6a).

The differences in the matrix organization generated by different concentrations of calcium were also highlighted from the SEM acquisition of DDS after the drug release; it was possible to observe a further reduction in height for the DDS0.05_5 (Figure 6d), while the matrix of DDS0.10_5 after dissolution and re-drying (Figure 6c) preserved the hollow filaments with an almost unchanged matrix and simply emptied of the drug.

### 2.6. Drug Content Analysis and Drug-Loading Efficiency

The information about the drug content and the drug loading efficiency, obtained by DDS dissolution in phosphate buffered saline (PBS) solution, was among the most interesting data related to the matrix composition and the drying behavior. For both batches, DDS0.05_R5 and DDS0.10_R5, the drug loading efficiency was extremely high (Table 1), close to 100% for the batch containing the highest amount of calcium, suggesting a direct relationship between the stiffness/structuration of the matrix and the load retention capacity during the drying. Further confirmation of the difference in drug loading capacity was obtained from the DC values, with a slight increase after the increase of calcium.

### 2.7. DDS Mechanical Strength

**Figure 7:**
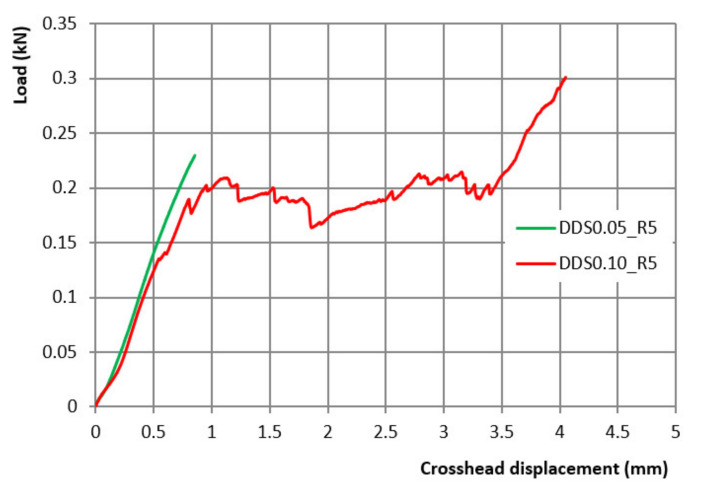
Load-crosshead curve of DDS0.05_R5 (green line) and DDS 0.10_R5 (red line).

The load curves (Figure 7) show the differences in the matrix behavior as the calcium chloride concentration varies, with a linear increase in the recorded strength, up to the structure crack for batch DDS0.05_R5 (green line) and a series of peaks corresponding to the progressive crushing of the different hollow filaments clearly distinguishable in the batch DDS 0.10_R5 (red line).

### 2.8. Buoyancy and In Vitro Drug Release

**Figure 8:**
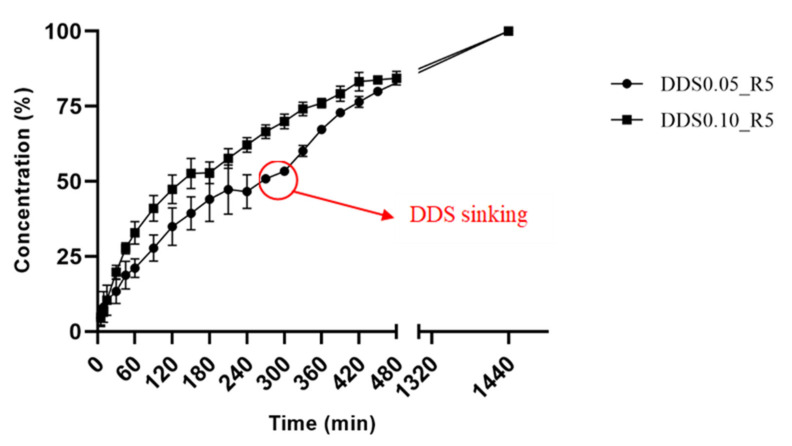
Dissolution profile of (●) DDS0.05_R5 and (■) DDS0.10_R5 (the *x*-axis is interrupted at 8 h and re-plotted at 22 h for better data display). The red arrow indicates the time interval in which the sinking of DDS0.05_R5 occurs.

Batches of DDS0.05_R5 showed a considerable variation in the buoyancy properties, with a lag phase in the range from 0 to 30 min, and a floating time about 5 h with a relative standard deviation higher than 10%. Differently, the DDS0.10_R5 batches were characterized by the absence of a lag phase and a high reproducibility in the floating time of about 14 h (14 ± 1). Trough the dissolution tests, for both DDS0.05_R5 and DDS0.10_R5 a complete drug release was reached before 24 h, even if after about 5 h the DDS0.05_R5 sank (Figure 8).

### 2.9. Release Kinetics Fitting Studies

As shown by Table 2, the Peppas–Korsmeyer equation fit the release data well, producing r2adj > 0.92 and lower values of reduced χ^2^ for all formulations. Higuchi’s model showed in both cases a lack of fit for the experimental points. The values of the n coefficient indicate a complex non-Fickian transport mechanism involving matrix erosion and swelling depending on the internal network of the matrix.

## 3. Discussion

The improvement in biopharmaceutical performance for a poorly soluble drug such as ricobendazole is strictly related to the enhancement of its pharmacokinetic properties [1]. To date, the dosage forms commercially available have been mainly acid solutions, which can cause adverse reactions [36]. It is therefore necessary to explore new innovative dosage forms that may lead to maximizing RBZ treatment efficacy.

In this work, a new RBZ floating system was produced using a pioneering co-axial semi-solid extrusion 3D printing technique previously developed by our research group [28]. This process provides the construction of a hollow DDS, exploiting the extemporaneous gelation of alginate with calcium during the extrusion of feeds: alginate ink, a sodium alginate solution, and crosslinking ink containing calcium ions as crosslinking agent, HEC as thickening agent, and RBZ as active ingredient. To develop this DDS loaded with ricobendazole, the first part of the work was focused on the optimization of the crosslinking ink, in accordance with both the physico-chemical characteristics of RBZ and the printing requirements of the ink (Table 3).

The alcohol content was fixed at 20% *v*/*v*, since its further increase did not improve gel extrudability, highlighting on the contrary HEC hydration issues. Once the optimal hydroalcoholic mixture was selected with the aim of investigating its role in both the printing and the drying phases of the production process as well as on the buoyancy and release properties of the final DDS, two different concentrations of calcium chloride were tested, which did not significantly influence the gel viscosity. To prevent the ink gels from spreading onto the build plate, which could negatively affect the printing resolution, the flow rates were optimized in accordance with their viscosity differences to obtain a complete gelation of the alginate during the extrusion and an excellent shape retention after printing (Figure 2, with the masking tape selected as print bed support material). In fact, from the rheological study, the ink feed showed a loss factor > 1, suggesting for this gel a clear prevalence of liquid behavior, which was also confirmed by the minimum decrease in the viscosity under an increased shear rate. On the other hand, all crosslinking feeds showed a loss factor of about 0.5, with comparable gel-like properties and shear thinning behavior.

The results of the second part of the research, dedicated to the printing of the DDS, showed that the different calcium concentrations did not influence the printing performance in terms of resolution and shape retention, but had important effects during the drying step, with a significant collapse of the matrix for the batch containing a lower amount of calcium—a sign of a lower organization of the calcium alginate matrix. The latter, clearly visible from the section of the DDS produced (Figure 6), then led to a different performance of the drug-loaded DDS in terms of mechanical strength, lag time, buoyancy, and ricobendazole release. In particular, #DDS0.05_R5, with a reduced empty space in the printed filaments after the drying process, showed a lag time, after which the formulations floated for only 5 h, compared to the 14 h of the DDS0.1_R5.

An important part of the research was dedicated to the in vitro evaluation of drug release from GRDDS, with the aim of comparing the release profiles of different batches and consequentially understanding the influence of the matrix composition on the drug release kinetics. Conventional test conditions (0.1 M HCl solution at pH 1.2 ± 0.5, without enzymes) selected to obtain reproducible data are in accordance with the technical procedure described in USP44 and have been widely reported and adopted in literature. However, they are undoubtedly far from mimicking the physiological conditions. Further in vivo studies will be needed to clarify the fate of the GRDDS after oral administration.

From the comparison between the drug release profiles, reported in Figure 5, it is possible to observe a significant variation in release rates due to the difference in the amount of calcium in the matrix according to the peculiar DDS properties previously described. This difference was also evidenced by the release kinetics fitting study. In fact, DDS0.10_R5 showed a lower n value, around 0.69, which is a sign of a tougher internal structure less prone to swelling compared to the external layers. In particular, the release data of DDS0.05_R5, recorded in the first two hours, showed higher standard deviation values of drug released compared to DDS0.10_R5, probably due to the different exposure of the DDS0.05_R5 surface to the dissolution medium during the inconstant lag phase.

## 4. Materials and Methods

### 4.1. Materials

All ingredients used for the preparations of the inks, both alginate and crosslinking, and the media for analysis are listed below: the Sodium Alginate European Pharmacopoeia 10th (CAS 9005-38-3, Carlo Erba, Milano, Italy) with a ratio between β-d-mannuronic:β-l-guluronic acid of 1.3, *M*_W_ > 200,000 g/mol, and 1% aqueous solutions viscosity 65 mPa·s was selected as the polymeric matrix; hydroxyethyl cellulose high viscosity (CAS 9004-62-0, ACEF, Piacenza, Italy) (2100 mP·s of 1% aqueous solutions), with a 1.5 degree of substitution was used as thickening agent; calcium chloride (CAS 10043-52-4, VWR International, Milano, Italy) was used as the crosslinking agent; Tween^®^ 85 (Tw, CAS 90005-70-3, Sigma-Aldrich, Milano, Italy) was used as stabilizer; ricobendazole (RBZ, CAS 54029-12-8, Todo Droga^®^ Córdoba, Argentina) ≥ 98% (HPLC) was used as the API focus of this study; ethanol 96% (CAS 64-17-5, Sigma-Aldrich, Milano, Italy) Reag. Ph Eur., hydrochloric acid 37% *w*/*w* (ACS reagent, CAS 7647-01-0, Sigma-Aldrich, Milano, Italy) and sodium phosphate (CAS 7601-54-9, Sigma-Aldrich, Milano, Italy) were used for the preparation of the dissolution media.

### 4.2. Methods

#### 4.2.1. Alginate and Crosslinking Ink Preparation

For the alginate ink preparation, 6% *w*/*v* of sodium alginate powder was added into a pre-settled volume of water and magnetically stirred at 70 rpm to reach full hydration of the matrix. The crosslinking ink containing the calcium chloride for the co-axial extrusion system [22] was optimized in accordance with the physico-chemical properties of ricobendazole, the active ingredient selected. In particular, to slightly improve the drug solubility (62 µg/mL in water and 1.2 mg/mL in ethanol [8]) and maintain the gel extrudability, a 80/0 water ethanol solution was used as solvent and the HEC was added at a concentration of 2.0% *w*/*v*. Firstly, the drug was dispersed into the ethanol and then the alcoholic dispersion was added to the aqueous calcium chloride solution to obtain an hydroalcoholic suspension (final concentration 5% *w*/*v* in RBZ; 0.05–0.1 M in CaCl_2_) stabilized with Tween 85 (0.1% *v*/*v*). The fine suspension was finally thickened with HEC (2.0% *w*/*v*) to obtain extrudable ink (Table 2).

#### 4.2.2. Rheological Study

With the aim of highlighting the gel properties and relating them to the printing requirements, two different rheological tests, i.e., rotational (with a shear rate from 1 × 1/s to 25 × 1/s) and amplitude sweep (with a shear strain from 0.1% to 100%), were carried out through an MCR 102 rheometer (Anton Paar, Rivoli, Italy). In particular, the instrument was equipped with a parallel plate (PP25, with a diameter of 24.985 mm) and the measuring gap value was set at 0.5 mm; the samples were loaded onto the plate through the same syringe used for the printing process and measured after a rest of 1 min at 25 ± 1 °C. All data were acquired and analyzed by RheoCompass™ Software (Anton Paar, Rivoli, Italy).

#### 4.2.3. Design, Development, and Production of DDS

The 3D digital model of RBZ DDS was developed via the CAD software Rhinoceros 6. In consideration of the size reduction during the drying, the 3D structure selected was a double circumference toroid with an external diameter of 22 mm, a height of 14 mm, and a central hole of 10 mm. The model, in the form of an STL file, was processed with Cura 4.6.1 (Ultimaker, Utrecht, The Netherlands) to optimize the printing parameters (initial layer height, 3 mm; layer height, 2.75 mm; number of layers, 5). The flow rate was fixed at 50 µL/min for the alginate ink and at 100 µL/min for the crosslinking feed.

#### 4.2.4. DSC Thermal Analysis

The drug–polymer interactions were investigated by differential scanning calorimetry of the ricobendazole raw material, the drug-free DDS, and the RBZ-loaded DDS in a dynamic configuration from 25 °C to 300 °C at 10 °C/min (DSC 822e, Mettler Toledo, Giessen, Germany). A total of 3–5 mg of each sample in the form of powder, or a DDS fragment, were weighted in a 40 µL predrilled aluminum pan, which was closed and pierced. The data were processed by the Mettler Toledo STARe software and reported as thermograms in the results section.

#### 4.2.5. FT-IR Analysis

FT-IR analysis was carried out using a FT-IR spectrophotometer (Spotlight 400N FT-NIR Imaging System, Perkin Elmer Inc., Walyham, MA, USA) equipped with an ATR accessory (ZnSe crystal plate) to detect any changes in the drug structure during the printing and drying processes. The powders and a dried film of the crosslinking feeds, as well as the printed formulations, were analyzed using 128 scans and a 1 cm^−1^ resolution step in the spectral range of 4000–600 cm^−1^.

#### 4.2.6. Morphological Analysis

For the morphological characterization: pictures of DDS were acquired by a single lens reflex camera (Canon EOS 600D); microscopic details of DDS structure were detected at different magnifications ×30, ×100, ×1000 by scanning electron microscopy (Tescan Solaris, Tescan Orsay Holding, Czech Republic) after metallization by LEICA EMSCD005, producing a 200–400 Å-thick gold layer.

#### 4.2.7. DDS Printing Reproducibility: Mass and Dimensional Analysis

The mass and the dimensions (diameter, D; and height, H), both after printing and after drying, of all batches produced were analyzed using an analytical balance and a digital caliper, respectively. In particular, the mass after printing (M_p_) in grams and the mass variation after drying (M_d_), expressed according to the following equation, were reported.

(1) $$M_{d}\;\left(\%\right)=\frac{\mathrm{DDS}\;\mathrm{Mass}\;\mathrm{after}\;\mathrm{drying}\;\left(\mathrm{mg}\right)}{\mathrm{DDS}\;\mathrm{Mass}\;\mathrm{after}\;\mathrm{printing}\;\left(\mathrm{mg}\right)}*100$$

Similarly, the variation in dimension after printing (D_p_ and H_p_, in relation to the digital model) and after drying (D_d_ and H_d_, in relation to the printed model) were calculated according to the following equations:

(2) $$D_{p}\;\left(\%\right)=\frac{D_{\mathrm{Printed}\;\mathrm{model}}}{D_{\mathrm{Digital}\;\mathrm{model}}}*100;$$

(3) $$H_{p}\;\left(\%\right)=\frac{H_{\mathrm{Printed}\;\mathrm{model}}}{H_{\mathrm{Digital}\;\mathrm{model}}}*100$$

(4) $$D_{d}\;\left(\%\right)=\frac{D_{\mathrm{Dried}\;\mathrm{model}}}{D_{\mathrm{Printed}\;\mathrm{model}}}*100$$

(5) $$H_{d}\;\left(\%\right)=\frac{H_{\mathrm{Dried}\;\mathrm{model}}}{H_{\mathrm{Printed}\;\mathrm{model}}}*100$$

#### 4.2.8. Drug Content Analysis and Drug-Loading Efficiency

The drug content (DC) and the drug loading efficiency (DLE) of the DDS were calculated in accordance with the equations given below:

(6) $$\mathrm{DC}\;\left(\%\right)=\frac{\mathrm{Mass}\;\mathrm{of}\;\mathrm{RBZ}\;\mathrm{detected}\;\left(\mathrm{mg}\right)}{\mathrm{Mass}\;\mathrm{of}\;\mathrm{RBZ}\;\mathrm{DDS}\;\left(\mathrm{mg}\right)}*100$$

and

(7) $$\mathrm{DLE}\;\left(\%\right)=\frac{\mathrm{Mass}\;\mathrm{of}\;\mathrm{RBZ}\;\mathrm{detected}\;\left(\mathrm{mg}\right)}{\mathrm{Mass}\;\mathrm{of}\;\mathrm{RBZ}\;\mathrm{predicted}\;\left(\mathrm{mg}\right)}*100$$

To carry out these experiments, at least three DDSs were solubilized in PBS (250 mL, pH 6.8). The RBZ content was obtained by relating the absorbance values of each sample at a 295 nm wavelength (λ) (detection acquired by using UV–vis Spectrophotometer, Evolution 201 UV–vis Spectrophotometer, Thermo Fischer Scientific Inc., Milano, Italy) with a PBS calibration curve (concentration range from 10.3 µg/mL to 63 µg/mL).

#### 4.2.9. DDS Mechanical Strength

The DDS mechanical strength analysis was conducted with a CMT 6000 dynamometer (MST^®^, Beijing, China) equipped with a 1 kN load cell. One DDS was placed between two steel plates; the upper one, connected with the load cell, was then manually lowered towards the DDS surface [37]. The test in the compression mode was then started at a velocity of 5 mm/min. The data were reported in a graph obtained plotting the recorded strength (kN) against the travel made by the upper plate (mm).

#### 4.2.10. Buoyancy and In Vitro Drug Release Evaluation

In vitro drug release was performed using USP dissolution Apparatus II (AT7 Smart Dissolution Tester, Sotax Corporation), with paddle configuration at 70 rpm stirring and 37 °C. In particular, the dried DDSs were placed in a pre-settled volume (750 mL) of dissolution medium, 0.1 M HCl solution at pH 1.2 ± 0.5, in accordance with the technical procedure described in USP44. For the drug release, 5 mL aliquots of dissolution medium were withdrawn at different intervals of time and replaced by a buffer (5′, 10′, 15′, 30′, 45′, 60′, 90′, 120′, 150′, 180′, 210′, 240′, 270′, 300′, 330′, 360′, 390′, 420′, 450′, 480′, and overnight). Each sample was appropriately diluted with dissolution medium to obtain a drug concentration in the range of the HCl calibration curve. For all samples, the results were shown in the form of a graph by specifying mean values and standard deviations for each point.

During the dissolution test, data about buoyancy properties, i.e., lag time before the buoyancy and the floating time, were obtained through visual evaluation and reported in minutes and hours, respectively.

#### 4.2.11. Release Kinetics Fitting Studies

Fitting analysis on the release data was performed by using two different kinetic models to clarify the release mechanism of the different formulations. In fact, both Higuchi’s model (Equation (8)) and the Peppas–Korsmeyer equation (Equation (9)) were used.

Higuchi’s model is one of the models most used for investigating pure Fickian transport when pure diffusion is the main driving force of the release [38]

(8) $$\mathrm{Higuchi}:{\;M}_{t}=A\sqrt{D\left(2C_{0}-C_{S}\right)t},$$

where M_t_ is the drug cumulative amount released at time t, t is time, A is the surface area, D the diffusivity of the drug through the matrix, and C_0_ and C_s_ are the initial drug concentration and drug solubility, respectively.

The Peppas–Korsmeyer equation [39,40] is used to mainly explain complex release mechanisms in which diffusion is coupled with erosion or swelling of the matrix:

(9) $$\mathrm{Peppas}-\mathrm{Korsmeyer}:\;\frac{M_{t}}{M_{\infty }}={\mathrm{kt}}^{n}$$

where M∞ is the drug amount released at infinity, k is a constant, and n is a diffusion coefficient which depends on the geometry of the system and on the release mechanism. When pure diffusion controls the release mechanism, n = 0.5, whereas when n = 1, the release mechanism is dominated by Case II transport, and Equation (9) turns into a zero-order kinetic.

The use of both equations is mainly limited to the description of the first 60% of the release. However, in this study the fitting performed over 60% of the release was compared to that over 100% of the profile to verify the adequacy of the procedure [41]. The correlation coefficient corrected for the degree of freedom of the system (r^2^_adj_), and reduced χ^2^ Levenberg–Marquardt methods for the minimization of the function were used to evaluate the lack of fit.

## 5. Conclusions

Drug delivery systems able to float while releasing the drug in simulated gastric fluids were successfully obtained by 3D printing semi-solid extrusion, exploiting the alginate ionotropic gelation immediately after ink extrusion. This gelation timing allowed for obtaining a calcium alginate filament containing the drug which was able to maintain the DDS shape after the printing process. Moreover, through the addition of the right amount of crosslinking agent, the matrix showed a proper stiffness to counteract the layer collapse during the drying process, with mechanical strength values comparable to conventional tablets.

The improved DDS was able to float with no lag time for about 14 h, releasing almost 80% of the loaded drug into the simulated gastric fluid during the floating time. Further in vivo studies will clarify whether this printed drug delivery system can be proposed as an interesting technological platform able to overcome ricobendazole pharmacokinetics issues, extending for many hours the contact time between the active compound and the infected tissue, as expected from an effective gastro-retentive system.

## Figures and Tables

**Table 1:** Mass and dimensional analysis, after printing and drying; drug content and drug loading efficiency of RBZ-loaded DDS.

Code	DDS0.05_R5	DDS0.10_R5
	After Printing	
M_p_ (g)	3.2 ± 0.2	3.2 ± 0.1
D_p_ (%)	110.2 ± 1.3	97.0 ± 2.7
H_p_ (%)	88.3 ± 2.8	104.7 ± 4.0
	After Drying	
M_d_ (%)	7.9 ± 0.4	8.7 ± 0.2
D_d_ (%)	78.7 ± 7.6	81.0 ± 2.2
H_d_ (%)	33.3 ± 10.7	55.0 ± 6.6

Drug Content (%)	34.8 ± 1.3	39.0 ± 2.1
Drug LoadingEfficiency (%)	81.9 ± 4.1	99.2 ± 2.4

M_p_, mass after printing; D_p_, diameter variation after printing (Equation (2)); H_p_, height variation after printing (Equation (3)); M_d_, mass variation after drying (Equation (1)); D_d_, diameter variation after drying (Equation (4)); H_d_, height variation after drying (Equation (5)).

**Table 2:** Fitting of different kinetic models on the release profiles of different formulations.

Code	Higuchi		Korsmeyer–Peppas		
	r^2^adj *	Reduced χ^2^ **^†^**	r^2^adj *	Reduced χ^2^ **^†^**	n (S.E.)
DDS0.05_R5	0.8615	7.83	0.9258	2.08	0.71 ± 0.08
DDS0.10_R5	0.8813	6.42	0.9135	2.52	0.69 ± 0.05

* r^2^adj: 1 − [(n − 1/n – k − 1)(1 − r^2^)], n: number of data points, k: number of independent variables. **^†^** χ^2^/DoF as obtained by the Levenberg-Marquardt method.

**Table 3:** Composition of both alginate and crosslinking inks.

Code	Inks			
DDS0.05_R5	Alginate		Crosslinking	
	Solvent		Solvent	
	H_2_O		H_2_O/EtOH 80/20 *v*/*v*	
	Ingredients		Ingredients	
	Sodium Alginate	6% *w*/*v*	CaCl_2_	0.05 M
			RBZ	5% *w*/*v*
			HEC	2% *w*/*v*
			Tw	0.1% *v*/*v*
**Code**	**Inks**			
DDS0.10_R5	Alginate		Crosslinking	
	Solvent		Solvent	
	H_2_0		H_2_O/EtOH 80/20 *v*/*v*	
	Ingredients		Ingredients	
	Sodium Alginate	6% *w*/*v*	CaCl_2_	0.10 M
			RBZ	5% *w*/*v*
			HEC	2% *w*/*v*
			Tw	0.1% *v*/*v*

## Data Availability

Not applicable.
